# Printed Microwave Metamaterial-Antenna Circuitries on Nickel Oxide Polymerized Palm Fiber Substrates

**DOI:** 10.1038/s41598-019-39736-8

**Published:** 2019-02-18

**Authors:** Taha A. Elwi

**Affiliations:** Department of Communication Engineering, Al-Mammon University College, Baghdad, Iraq

## Abstract

In this paper, the novelty of exploring the applications of the Iraqi Palm Tree Remnants (IPTR) mixed with Nickel Oxide Nanoparticles (NONP) hosted in Polyethylene (PE), called INP substrates, is utilized by printing metamaterial (MTM) based high gain microwave antennas on them. The proposed INP substrates are mainly created from pressed flexible organic fibers to suite the ink jet printing technologies. The complex relative constitutive parameters are characterized in terms of permittivity (ε) and permeability (μ) within the frequency range from 2 GHz up to 6 GHz using an open end dielectric probe and a T-stub transmission line technique. To validate the feasibility of the INP substrates, a very fine antenna structure of based a miniaturized Hilbert MTM based dipoles is printed on. A material printer with Sliver Nanoparticles Conductive Ink (SNPCI) is used to print the antenna structure. Commercial software packages, CST Microwave Studio (MWS) and Ansys High Frequency Structure Simulator (HFSS), are used to simulate the proposed antenna based on the measured constitutive parameters. A negligible difference is found between the measured and simulated results. Finally, an attractive effect on the retrieved constitutive parameters of the proposed MTM is found due to the proposed INP substrate.

## Introduction

Since their introduction in the 1960’s, MTM structures have been extensively investigated to enhance the antennas performance due to their untraditional electromagnetic properties^1^. For example, the antenna performance was enhanced using MTM structures based on a partial ground plane with electromagnetic band gap defects^2^. In^3^ and^4^, the antenna gain was improved using meta-surface as superstrates on the top of traditional antennas without considering the bandwidth enhancement or size reduction. The coupling reduction was improved significantly between adjacent antennas in their arrays after introducing the MTM structures within a limited size as in^5^ and^6^. A probe feed MTM antenna of enhanced bandwidth was proposed in^7^ based on circular array to exhibit circular polarized antenna radiations. Later, a polarization manipulation was inspired the authors in^8^ to realize a strategy to construct MTM based complementary transmissive ultra-thin meta-deflectors. In^9^, a Ku-band dual-circularly polarized broadside-beam MTM antenna was proposed based synthesizing subwavelength elliptical slotted metallic patches mounted on a grounded substrate. However, in^10^, the authors proposed a conformal wearable antenna for medical applications backed with a truncated MTM for radiation isolation.

Nanomaterials are also introduced as a next frontier in the wireless technologies^11^ including enhancing the antenna performance. In^12^, carbon nanotube ink was introduced to print different antennas on different substrates. The copper nanorods based on vertical arrays were grown on a microstrip patch to enhance their band and size reduction as proven in^13^. The possibility of printing antenna patches on solid substrates using SNPCI was investigated in^14^ for different wireless communication systems.

However, all the previous presented researches conducted their studies to MTM inclusions and/or nanomaterials without combining them together on organic fibers based substrates for patch antenna applications. As well as, introducing the nano-scale to the dielectric material based on organic substantial to create new substrates has not been considered yet in the new generations of the patch antenna structures. Therefore, a novel use of organic fibers based substrates combined to nanostructures for the applications of MTMs based microstrip patch antennas in this paper.

This decade, organic substrates based on plants fruits, leaves, stems, flowers, and fibers have attracted the substantial attentions of different industrial and scientific communities in the aspect of recycling logic to replace the old fashion substrates^15^. Most wet organic materials have the same amount of fibers to water ratio in terms of cellulose and hemicelluloses contents^16^. Therefore, the ability of creating adhesive substrates impacts efficiently the frame of this research. However, due to the mechanical difficulty of having a compacted hard reinforced composite fiber layer as a one piece, adding reactive materials such as polymers may effect on several parameters including the intrinsic properties matrix, melting temperature, and the strength of the fiber ligament^17^. There are several antenna parameters may rely on the method of reinforcing the fiber filling specially when mixed with other composites. Furthermore, such composites are highly recommended for the use in electromagnetic applications including the antenna structures.

It is well known that, for example, the conventional dipole antennas exhibit a relatively small bandwidth and a fixed gain which limit their use in modern wireless communication systems^18^. Therefore, the printed dipole antenna performance enhancements are taken place in this study by conducting both of nanomaterials and MTMs in nontraditional substrates made artificially of INP layers. A MTM array based on a Hilbert curve fractal unit cell printed with SNPCI on the INP substrate. The MTM structure is excited with two conventional dipoles of copper strips mounted on the INP substrate and resonant at the Wi-Fi bands. The concept of the established antenna manufacturing is conducted under atmospheric conditions. The addressed key issues in this paper are: Exploring the best manufacturing process to prepare the INP substrates and the printing process on them. Reduce the conductor loss effects of the SNPCI printout on the antenna performance in comparison to the traditional bulk materials of manufacturing. Realize the main effects of the prepared substrate on the antenna performance. Finally, the simulated results against measured values would be compared and discussed.

## Antenna Geometry and Considerations

**Figure 1:**
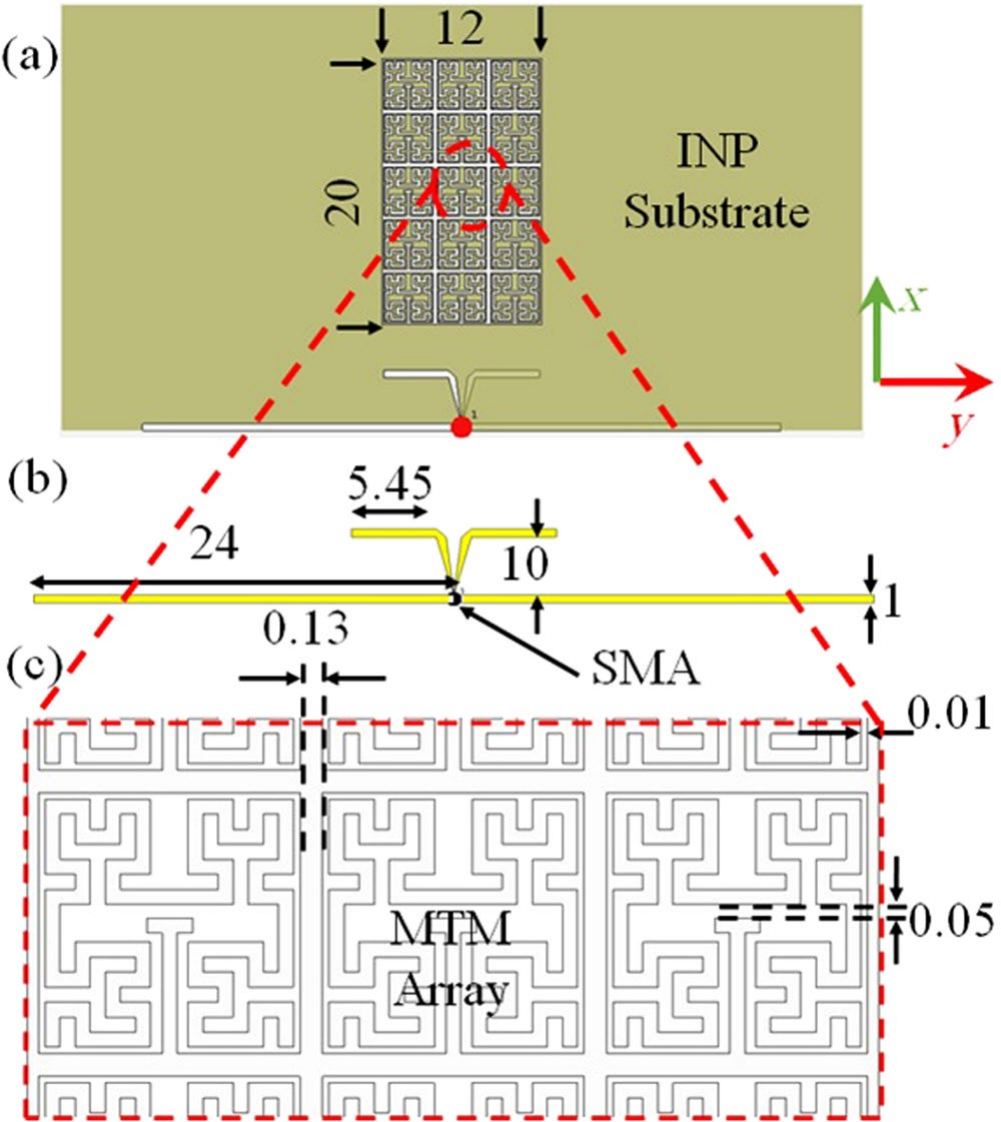
Geometry of the proposed antenna; (**a**) Front view, (**b**) Dipoles structure (**c**) Magnified picture from the MTM structure. Note: All the presented dimensions are in mm scale.

The antenna geometry, see Fig. 1, is consistent of an array of 3 × 5 planar Hilbert elements printed on the INP substrate of 0.8 mm thickness. A fractal MTM design is chosen to reduce the effective area to a 4 × 4 mm^2^ and provide multiple frequency bands excited by a stub capacitive coupling. Due to the fact that the electrical conductivity has a significant effect on the total antenna radiation efficiency, it is decided in this paper to minimize the printed conductor traces as possible to avoid the side effects of conduction loss. In fact, the SNPCI is a material with a limited conductivity, about 8.8 × 10^4^ S/m^19^, which exhibits a significant conduction loss. Moreover, increasing the printed trace width may increase the amount of dielectric losses^20^ as will be seen later. In such case, minimizing the effective printed conductive area is provided with such geometry to avoid the dielectric loss due to the substrate material. Such design consideration is studied later in this paper to show the effect of the conductor trace width on the total losses. Nevertheless, the Hilbert line width is chosen to avoid the unwanted cross-lines between the inner and outer traces according to the manufacturing processes limitations. The radiation element is introduced as a printed dipole antenna resonant at 2.45 GHz and 5.8 GHz to excite the MTM Hilbert surface.

Now, the individual unit cell is consistent of a stub resonator coupled to the 3^rd^ Hilbert-fractal geometry framed with a close loop square ring. The main contributions of the proposed MTM structure are the following: The Hilbert structure provides a significant MTM size reduction^1^. The stub resonator couples the energy to the Hilbert structure trough a capacitive resonance. While, the closed ring resonator inductively matches the energy with the Hilbert stature. For only the Hilbert structure, the perimeter (*p*) and the center frequency *f*_Hilbert_ can be determined using the following equations:

1 $$p=\frac{{4}^{n+1}-1}{{2}^{n+1}-1}l$$

2 $${f}_{\mathrm{Hilbert}}=\frac{{\rm{c}}}{2p\sqrt{{{\rm{\varepsilon }}}_{{\rm{e}}}}}$$

where, *n* is the number of the iterations, *l* is the Hilbert side length, *ε*_e_ is the effective permittivity. While, the resonance of the stub resonator occurs at

3 $${f}_{stub}=\frac{m\,c}{4{l}_{stub}\sqrt{{{\rm{\varepsilon }}}_{{\rm{e}}}}}$$

where, *l*_stub_ is the stub length and *m* is a multiple integer. Moreover the resonance of the closed loop ring can be obtained from

4 $${f}_{ring}=\frac{m\,c}{4{l}_{ring}\sqrt{{{\rm{\varepsilon }}}_{{\rm{e}}}}}$$

where, *l*_*ring*_ is the side length. Therefore, all the parameters in equations (2, 3 and 4) must agree to provide the same frequency resonance at which *f*_Hilbert_ = *f*_stub_ = *f*_ring_. In this case, the individual unit cell may provide resonances around the multiples of 2.45 GHz to suite the Wi-Fi bands.

**Figure 2:**
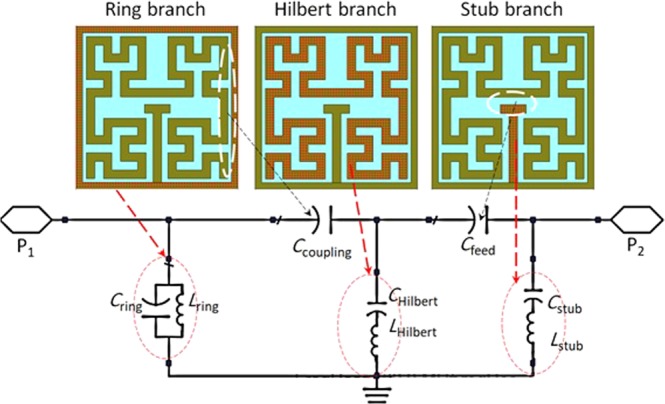
Equivalent circuit diagram based on the disassembled MTM unit cell.

Now, the equivalent circuit diagram of the unit cell that explains the function of the MTM is presented in Fig. 2. As seen in Fig. 2, the unit cell is disassembled to three parts that presented by inductor-capacitor branches. The closed square ring is coupled to the Hilbert branch through a coupling feed capacitor (*C*_coupling_). Then, the stub resonator branch is capacitive coupling to the Hilbert branch trough *C*_Feed_.

## Fabrication Process and Electromagnetic Properties Characterizations

### Substrate preparation

To prepare the INP substrates, the powder of IPTR must be prepared first from raw materials. Therefore, IPTR are selected where very rich with fibers^21^. Next, IPTR are crushed to a micro powder to be washed with worm water, 100 °C, to remove all dust and surface wax for several times. The floating powder that is considered the less material density leftover would be collected for the prepared substrates. The collected leftover must be dried for two hours using a convection oven, than washed with acetone to remove any clustered wax due to drying process. By using a mechanical vibration filter, the collected powder is separated to 100 µm size at maximum.

Now, the mixture ratios of the prepared samples are classified to three cases as listed in Table 1. The prepared samples are mixed using a Thermo Haake blending machine after heating up the mixture to 180 °C with 50 rpm rotation for 30 minutes. The substrate of 0.8 mm thickness is prepared by pressing the prepared texture inside a 10 × 8 cm^2^ mold. The prepared texture inside the mold is heated up to 100 °C then pressed with a high pressure for ten minutes. The applied pressure has a significant impact on the material density in which the electromagnetic and mechanical properties may change with the pressure change. Therefore, the author is decided to change the pressure from 90 to 115 to 130 kg/cm^2^ on the two sides to compact a slim substrate layer. Nevertheless, the change of the prepared samples weight with respect to the pressure change is listed in Table 1.

**Table 1:** Prepared sample surface properties measurements.

IPTR	NONP	PE	Pressure kg/cm2	*α*(deg.)	*R*_a_(µm)	Weight (g)	No.
3.4	0	6.5	90	88	0.25	5.608	A1
			115	79	0.22	7.107	A2
			130	72	0.19	9.391	A3
0	0.1	6.5	90	125	0.18	5.509	B4
			115	119	0.16	6.921	B5
			130	101	0.13	9.611	B6
3.4	0.1	6.5	90	70	0.10	6.126	C7
			115	66	0.09	7.833	C8
			130	58	0.07	11.218	C9

The mechanical properties of the prepared samples texture in terms of surface roughness change (*R*_a_) and contact angle (*α*). These two factors are considered the effective measure of wettability (sticky) and adhesiveness (self-adhesive) that are given by *R*_a_ and *α*, respectively. The measurements are conducted to those three samples and listed in Table 1. The contact angle is measured using Optical Tensiometers of DYNE technology, while, the surface roughness change of the prepared texture is measured using portable microscan LaserCheck equipment from Schmitt industries, Inc. After applying the surface measurements, the author used a five digit sensitive digital scale to weight the prepared samples as listed in Table 1. Such measurement shows the change of the relative material density that has a significant impact on the effective electromagnetic properties of the material^21^.

Now, the author decided to select the sample number C9 of the best wettability and highest adhesiveness to apply the surface plasma treatment based oxygen to reduce the surface tension further more for the selected sample. Such treatment is involved to increase the surface wettability that becomes more adhesive to the printing process^12^. Therefore, to prepare polymerized flexible substrates for printing, the initial treatment by oxygen plasma is conducted for 2 minutes in which a high degree of hydrophilicity is induced that increase SNPCI adhesion. This treatment is performed inside a cylindrical cavity in series with rotary pump and an RF source. The cavity internal pressure is set to 0.002Torr before the oxygen introduction. This process is carried out at 0.2 kW under an oxygen flow pressure of 0.4Torr.

### Substrate characterization

**Figure 3:**
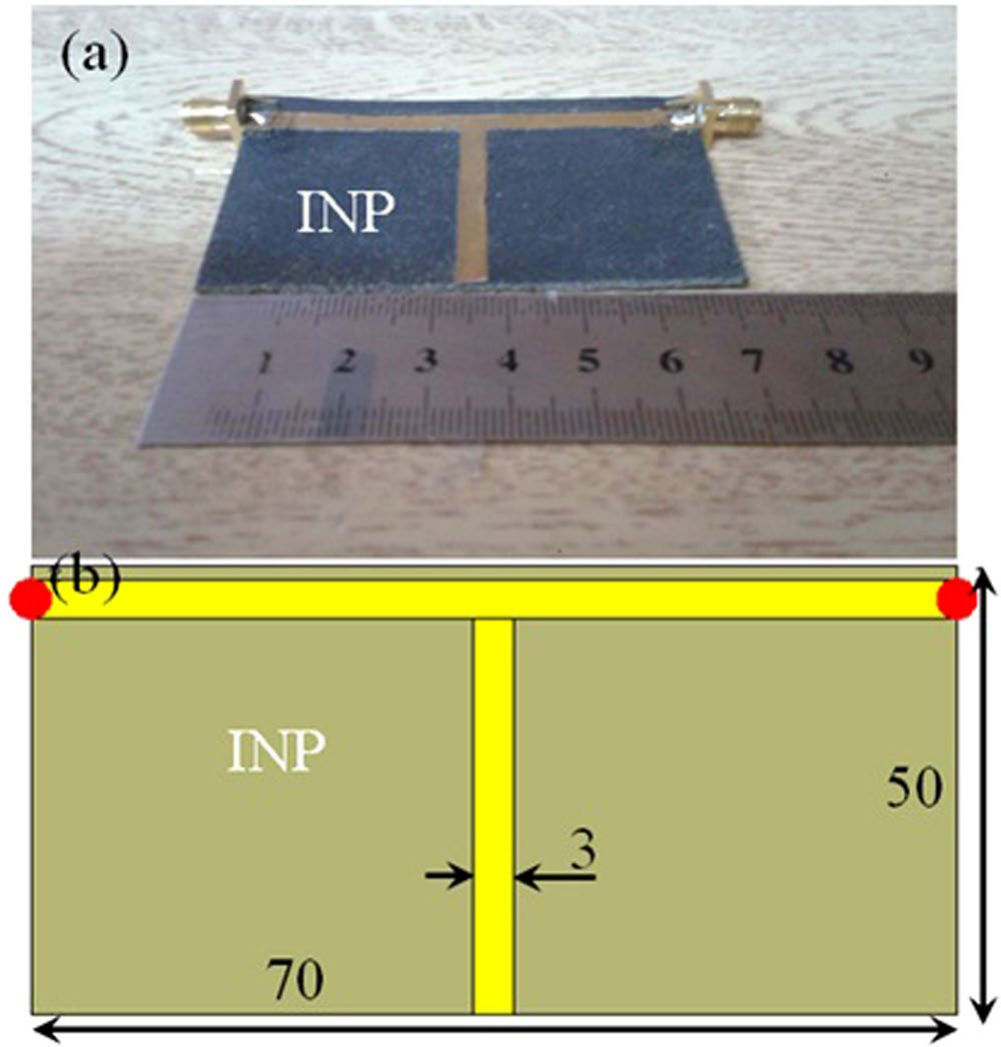
Prepared INP substrate-based T-stub resonator; (**a**) Fabricated structure and (**b**) Simulated structure. Note: All the presented dimensions are in mm scale.

The T-stub resonator, see Fig. 3, based on copper is designed to resonant around the Wi-Fi bands for the purpose of ensuring the constitutive parameters measurement precision; that can be applied later to the antenna design for the Wi-Fi applications. The T-stub resonator is mounted on the INP substrate and fed with 50 Ω SMA connectors to measure the S-parameters. The INP substrate is backed with a copper ground plane.

**Figure 4:**
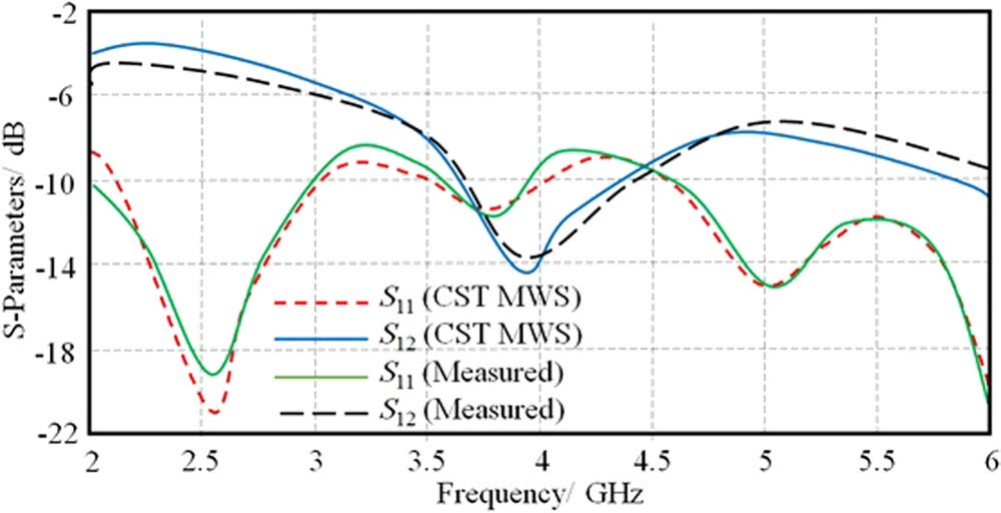
Measured S-parameters spectra of the prepared substrate.

Finally, the S-parameters are measured in terms of *S*_11_ and *S*_12_ after applying the port end and through line calibrations using HP 8720A VNA. Nicholson-Ross-Weir (NRW) technique is invoked to evaluate *ε*_r_ and *µ*_r_ values. Later on, an open coaxial Agilent E5071B probe is used to measure *ε*_r_ only for validation. Next, the same T-stub resonator is simulated inside CST MWS environment^19^ using the obtained *ε*_r_ and *µ*_r_ values from measurements, to compare the numerical results against the measured S-parameters as seen in Fig. 4. Therefore, the obtained *ε*_r_ and *µ*_r_ values are found to be about 3.106-j0.0314 and 1.548-j0.0907, respectively.

**Figure 5:**
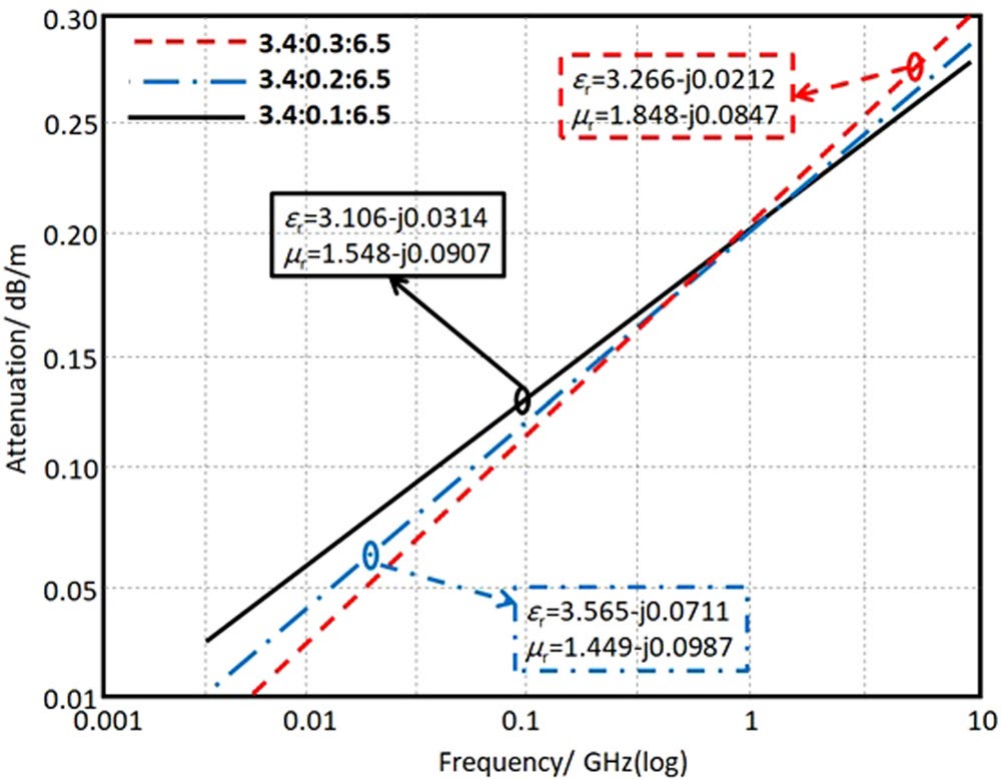
Attenuation spectra in log-log scale according to the retrieved *ε*_r_ and *μ*_r_ of the prepared substrate with different NONP ratios.

The dielectric attenuation factor (*α*_d_) of the used substrate is calculated using equation (5) in dB from^20^ and presented in Fig. 5.

5 $${\alpha }_{d}=27.3\,[(\frac{{\varepsilon }_{e}-1}{{\varepsilon }_{r}-1})(\frac{{\varepsilon }_{r}}{{\varepsilon }_{e}})(\frac{tan\,{\delta }_{e}}{{\lambda }_{g}})+(\frac{{\mu }_{e}-1}{{\mu }_{r}-1})(\frac{{\mu }_{r}}{{\mu }_{e}})(\frac{tan\,{\delta }_{m}}{{\lambda }_{g}})]$$

where, *ε*_e_ and *μ*_e_ are the effective permittivity and permeability, respectively. While, tan*δ*_e_ and tan*δ*_m_ are the electrical and magnetic loss tangents of the prepared substrate, respectively. Figure 5 shows the attenuation change with the frequency band of interest for different NONP ratios in the prepared substrate. It is found that the attenuation increases significantly with the frequency increase. Nevertheless, according to the measured *ε* and *µ* values, the attenuation curves are significantly changed due to NONP increase, i.e. an observable change may happen in the attention spectra of the prepared substrate by changing the particular ratio from 0.1, 0.2, to 0.3 as depicted in Fig. 5.

### MTM characterizations

The proposed MTM unit cell is constructed based on the 3^rd^ order of the Hilbert fractal geometry. Such structure increases the effective electrical length within a limited area excited by a capacitive coupling using a stub structure. The center frequency of the proposed unit cell is given by:

6 $${f}_{0}=\frac{(cw/l)}{p\sqrt{{{\epsilon }}_{re}}}$$

where; *w* is the substrate thickness, *l* is the unit cell side length, *p* is the unit cell perimeter. Based on the calculated frequency resonances from equations (2, 3 and 4), the circuit elements in Fig. 2 are calculated based on mutual inductance method reported in^22^ as listed in Table 2.

**Table 2:** Equivalent circuit lumped elements.

Circuit Elements	Value
*C*_ring_	0.52 fF
*L*_ring_	0.11 nH
*C*_Hilbert_	0.16 pF
*L*_Hilbert_	1.32 µH
*C*_stub_	0.01 pF
*L*_stub_	0.12 µH
*C*_coupling_	4.31 fF
*C*_feed_	34.1 fF

**Figure 6:**
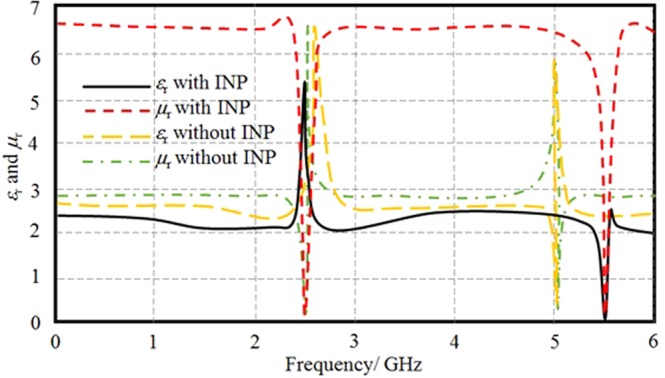
Retrieved effective *ε*_r_ and *μ*_r_ of the prepared unit cell with and without INP substrate. Note: Without INP substrate is based on air.

The electromagnetic properties of the proposed unit cell are retrieved inside an air box waveguide and carried within a numerical simulation based on CST MWS formulations to extract the S-parameters^14^. The effective complex *ε*_r_ and *µ*_r_ spectra are retrieved from the simulated S-parameters using the modified Nicolson Ross Weir^15^ and presented in Fig. 6 with and without the proposed substrate. It is found that the proposed unit cell based on the prepared substrate shows no −*ε*_r_ and/or −*μ*_r_ at any frequency within the band of interest. However, the unit cell shows almost zero *ε*_r_ and/or *μ*_r_ at 2.5 GHz and 5.8 GHz that may focus the radiation at these two bands. Nevertheless, the proposed unit cell shows different *ε*_r_ and *µ*_r_ spectra when it is mounted on the proposed substrate than its identical when it is mounted on air. Therefore, it is concluded that the proposed substrate has a significant effect on *ε*_r_ and *µ*_r_ spectra. On top of that, a frequency shift is achieved at the second band, 5.8 GHz, due to the introduction of INP substrate.

**Figure 7:**
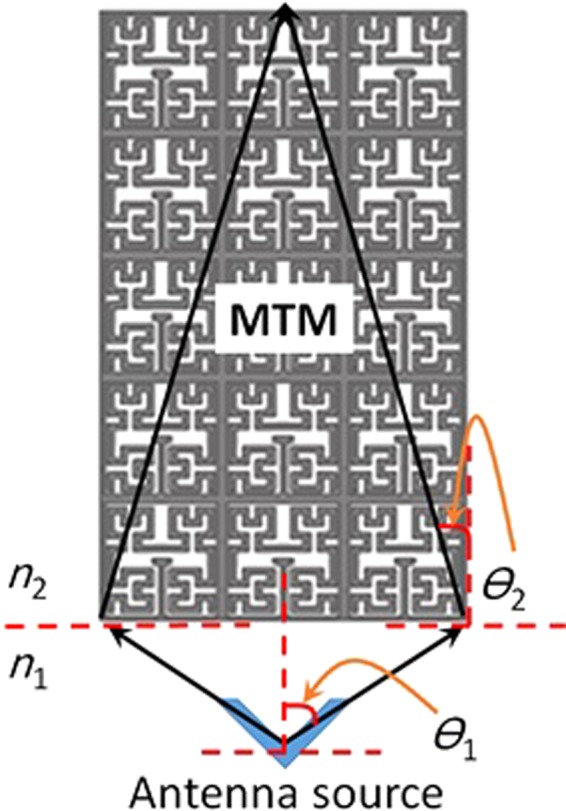
Ideal ray tracing of the emerging beam focusing from the antenna radiations.

To describe the function of the proposed MTM, let’s start from the retrieved electromagnetic properties in terms of the refractive index $n=\sqrt{{\varepsilon }_{r}{{\rm{\mu }}}_{r}}$ applied in Snell’s Law. As seen in Fig. 7, an ideal ray tracing model is presented based on Snell’s law according to the refractive index. In this design, the achieved reflective index over the entire band is found about n = 3.75. In the proposed analyses, it is assumed that the MTM refractive index is *n*_2_ = *n* = 3.75 with an emerged refection beam angle from the MTM side is *Ɵ*_2_. Next, *n*_1_ at the antenna side is assumed the same *n* of the INP substrate to be about 2.2 and an emerged beam from the antenna side is *Ɵ*_1_. Next, a parametric study will be applied later to setup the best antenna location with respect to the MTM array. Based on that, the radiation beam can be focused at a specific direction with a fixed distance to avoid the critical angle of incidence^23^. In such case, the angle of refraction of the emerged beam from the MTM side is found to be 17.5°; in which, the gain can be maximized significantly as will be observed later.

### MTM and dipole antenna structures fabrication

SNPCI is printed with Ink-jet printing technology (IJPT) were recently applied to manufacture microwave devices due to their high resolution, low thermal process, very high processing speed, and the ability to print on flexible substrates^17^. DMP-2800 Dimatix FujiFilm printer is used to printout MTM and the dipoles structures on the prepared substrate, with 10 pl nominal drop re-filed cartridge. The substrate is placed on a vacuumed heated platen of 200 × 300 mm printing area. The printing process is monitored with a fiducial camera. The printing process is performed at 60 °C temperature. The same MTM pattern is re-printed for three times with 10 minutes delay between each layer. Such process is performed to reach the optimum conductivity with enough thickness, about 1 μm, to avoid the skin depth effects at the frequency band of interest.

After accomplishing the printing process, an annealing progression inside a ProtoFlow LPKF’s convection oven is curried out on the printed surface to avoid oxidization^18^. Nevertheless, the annealing process is invoked to avoid the surface roughness due to the gaps between the nanoparticles^24^. The annealing temperature is fixed at 100 °C for 12 hours to ensure good percolation channels by diminishing potential crakes due to glomeration of the nano structures that lead to conductivity reduction due to the surface roughness as depicted in Fig. 7(b). The SEM image in Fig. 7(b) shows a corresponded standard deviation of the measured surface profile about 100 nm. These images are obtained from the field emission Scanning Electron Microscope SEM (7000F JEOL Ltd., Tokyo, Japan) with 15 k accelerating voltage.

A four-probe conductivity measurement technique is invoked to measure the electrical conductivity of the prepared samples^17^. The electrical conductivity measurement is performed using Keithley 224 programmable current source and a Keithley 617 programmable electrometer. It is found that the electrical conductivity for the printed structure is about 8.8 × 10^4^ S/m that is applied in CST MWS and HFSS^25^ simulations. Such conductivity is achieved when the printout is based on 3 layers as presented in Table 3. This is due to the nanoparticles glomeration with increasing the printout thickness^14^. Such glomeration my generate crossover printed lines for the proposed MTM structure.

**Table 3:** Measured SNP printout conductivity.

No. of layer	Sample Thickness (µm)	Conductivity S/m
1	0.66	8.7 × 10^4^
2	0.75	8.6 × 10^4^
3	0.87	8.8 × 10^4^
4	1.11	8.5 × 10^4^
5	1.31	8.4 × 10^4^

**Figure 8:**
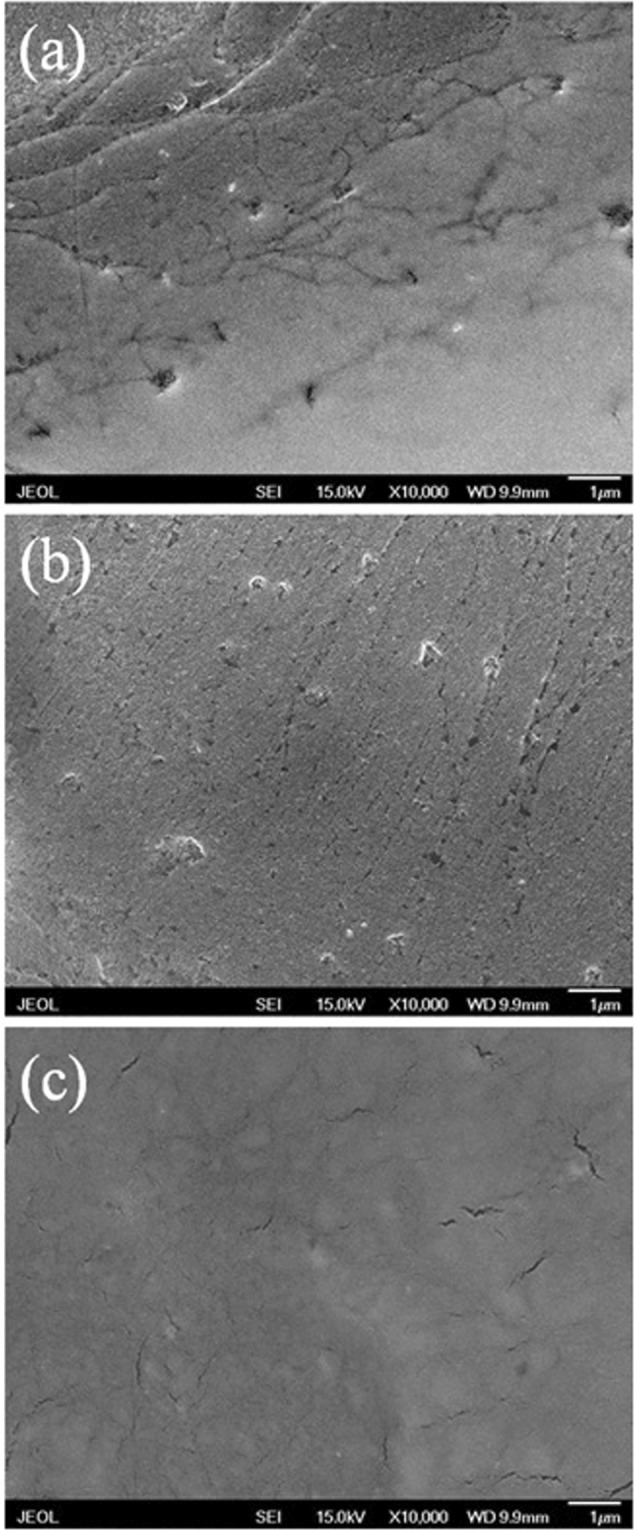
SEM images of the printed samples; (**a**) A3 substrate, (**b**) B6 substrate, and (**c**) C9 substrate.

Next, for these measurements, three samples are re-printed out with 3 layers only on three prepared substrates, A3, B6, and C9, to realize the effect of mixture ratio changing on the resolution printout and free electron channel percolations. Each sample is printed on a shape of a trace with a width of 0.5 mm. Images of the printed SNP traces on the three prepared substrates are presented in Fig. 8. It is found that the printout with 3 layers on sample number C9 shows the best resolution with best channel percolations; such observation is attributed to the fact by introducing the NONP additions to such substrate, the distribution of nanoparticles glomeration flatten significantly increases; this fact is attributed to the quantum nanoparticle gravitation^26^. Therefore, the author decided to construct the antenna on the substrate number C9 that shows the best printout flatten and homogeneity.

**Figure 9:**
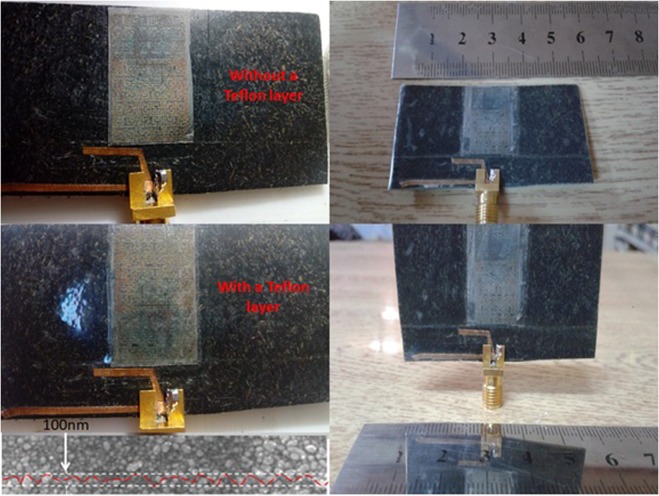
Manufactured prototype and SEM image of the printed parts.

After that, the dipoles are fabricated from a copper tape of 6 μm mounted on the proposed substrate from each side according to the numerical dimensions in Fig. 1. For further protection, a transparent Teflon layer, 1 μm thickness with almost *ε*_r_ = 1.098, is mounted on the whole structure accept the place at which the SMA port is soldered to the antenna as seen in Fig. 9. A preheating platen is used to press the entire structure for 1 minute with less than 5 kg/cm^2^ pressure on each side to remove any air gap papule formations.

**Figure 10:**
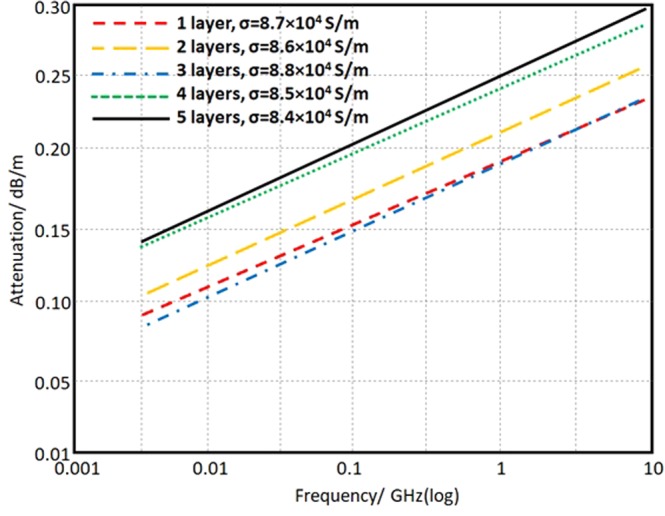
Conductor attenuation spectra in log-log scale according to the measured conductivity with different layers.

The conductor loss (*α*_c_), is the measure of the attenuation factor due to the conductive part in the antenna structure. However, this attenuation can be reduced significantly with the conductor width reduction^20^ as given in the following equation:

7 $${\alpha }_{c}=8.686\,[(\sqrt{\frac{\pi f\mu }{\sigma }})(\frac{1}{{Z}_{c}w})]$$

where, *µ*, *σ*, and *Z*_c_ are the permeability of the conductor, conductivity, and the characteristics impedance of the printed trace. While, *w* is the printed conductive trace width. The effect of changing the number of the printed layers on the measured conductivity that has an effect on the conductor loss is recorded in Fig. 10. It is found that the effect of changing the layer number has inconsistent effect on losses; this could be attributed to the surface roughness.

**Figure 11:**
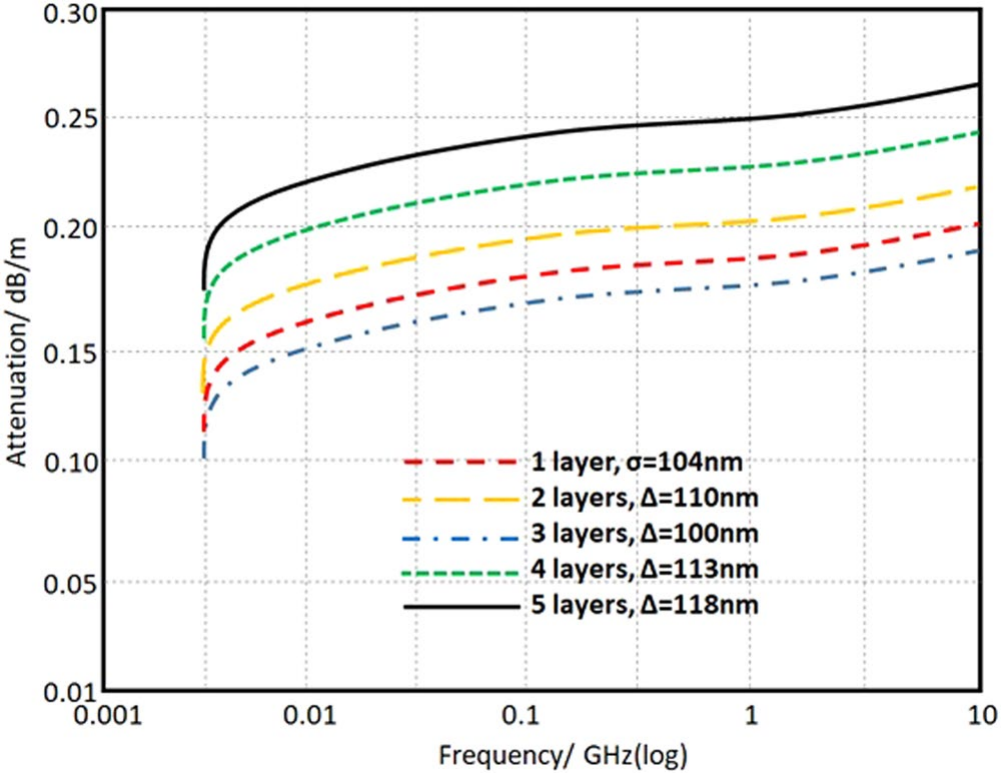
Conductor surface roughness attenuation in log-log scale according to the measured conductivity.

The conductor surface roughness loss (*α*_roughness_), is the measure of the signal attenuation due to the surface roughness of the conductor. Such attenuation maybe reduced by increasing the conductor thickness^20^ as given in the following equation:

8 $${\alpha }_{rouphness}=8.686{\alpha }_{c}\{1+\frac{2}{\pi }{{\tan }}^{-1}[1.4\frac{{\Delta }^{2}}{2}\mu \sigma \omega ]\}$$

this, Δ is the surface roughness, where, it is about 100 nm for the printed structure. The roughness loss spectrum is presented in Fig. 11. The obtained results from Fig. 10 emphasize the effect of the surface roughness on the electrical conductivity that is significantly affected by the number of the printed layer. Nevertheless, the obtained results in Fig. 11 agree logically with those presented in Fig. 10.

## Antenna Performance

**Figure 12:**
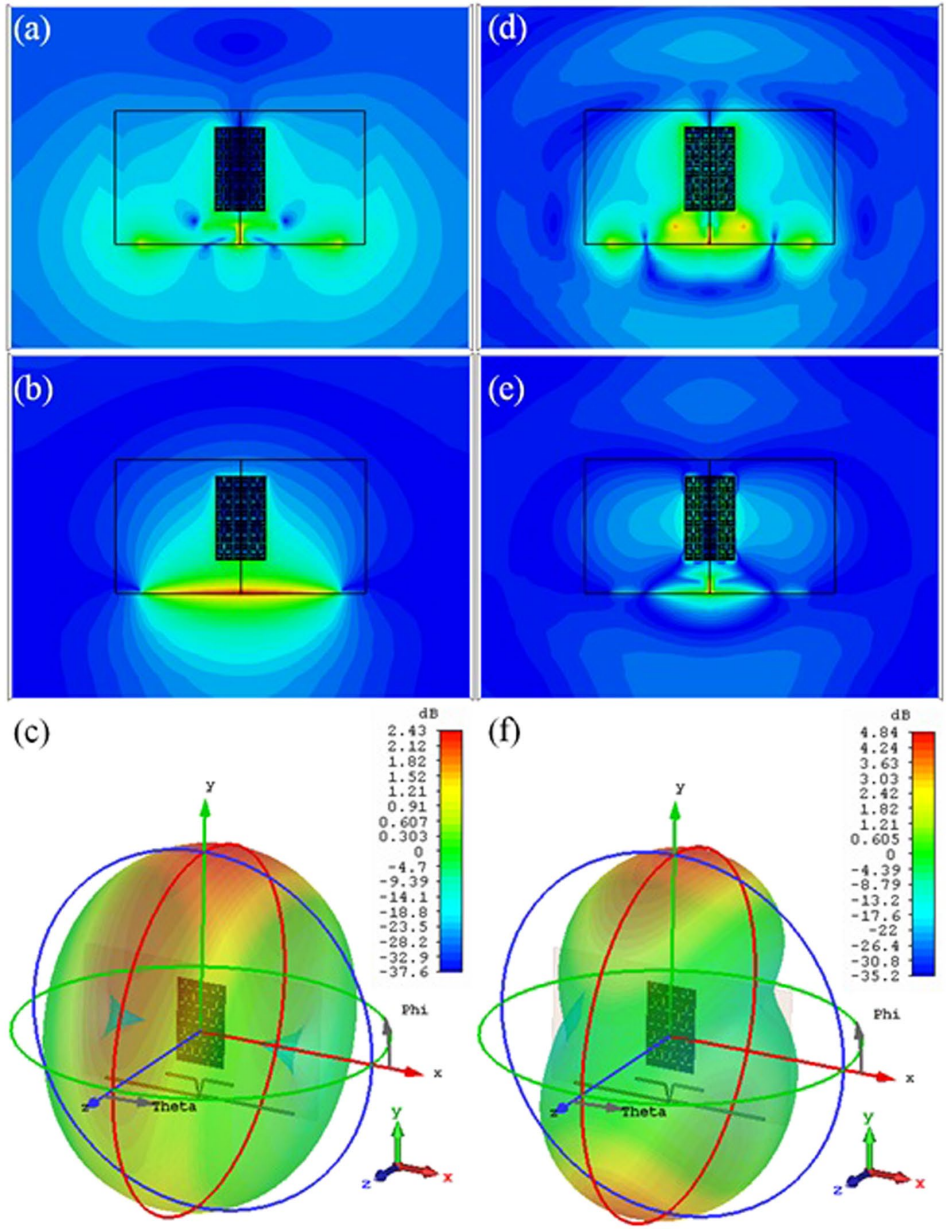
Antenna performance; (**a**) E-Field, (**b**) H-Field, and (**c**) 3-D Radiation Pattern at 2.45 GHz, (**d**) E-Field, (**e**) H-Field, and (**f**) 3-D Radiation Pattern at 5.8 GHz.

Now, the antenna performance using CST MWS numerical simulations is characterized in terms of *E*- and *H*-Fields with the 3-D radiation patterns at 2.45 GHz and 5.8 GHz as presented in Fig. 12. It is found that, the proposed MTM array focuses the radiated E- and H-fields from the antenna dipoles to the end-fire as seen in Fig. 12(a,b,d,e). Therefore, it is found that the antenna shows bore-sight gain values of 2.43 dBi and 4.83 dBi at 2.45 GHz and 5.8 GHz, respectively, as seen in Fig. 12(c,f).

**Figure 13:**
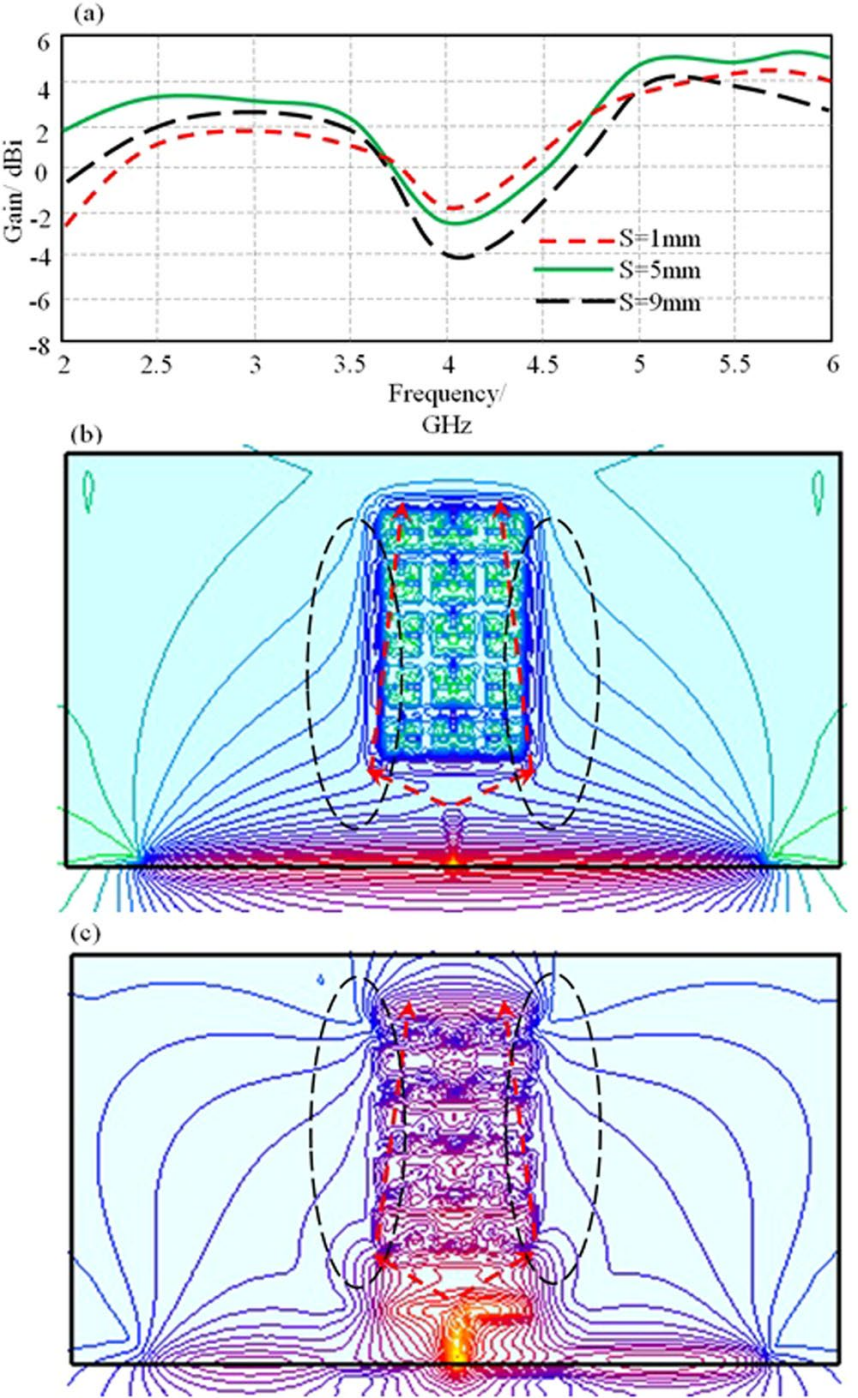
Antenna performance; (**a**) Gain spectrum change with different antenna location from MTM, (**b**) 2-D field focusing at 2.45 GHz, and (**c**) 2-D field focusing at 5.8 GHz. Note: The refracted beams are pointed out inside the dashed ellipses.

**Figure 14:**
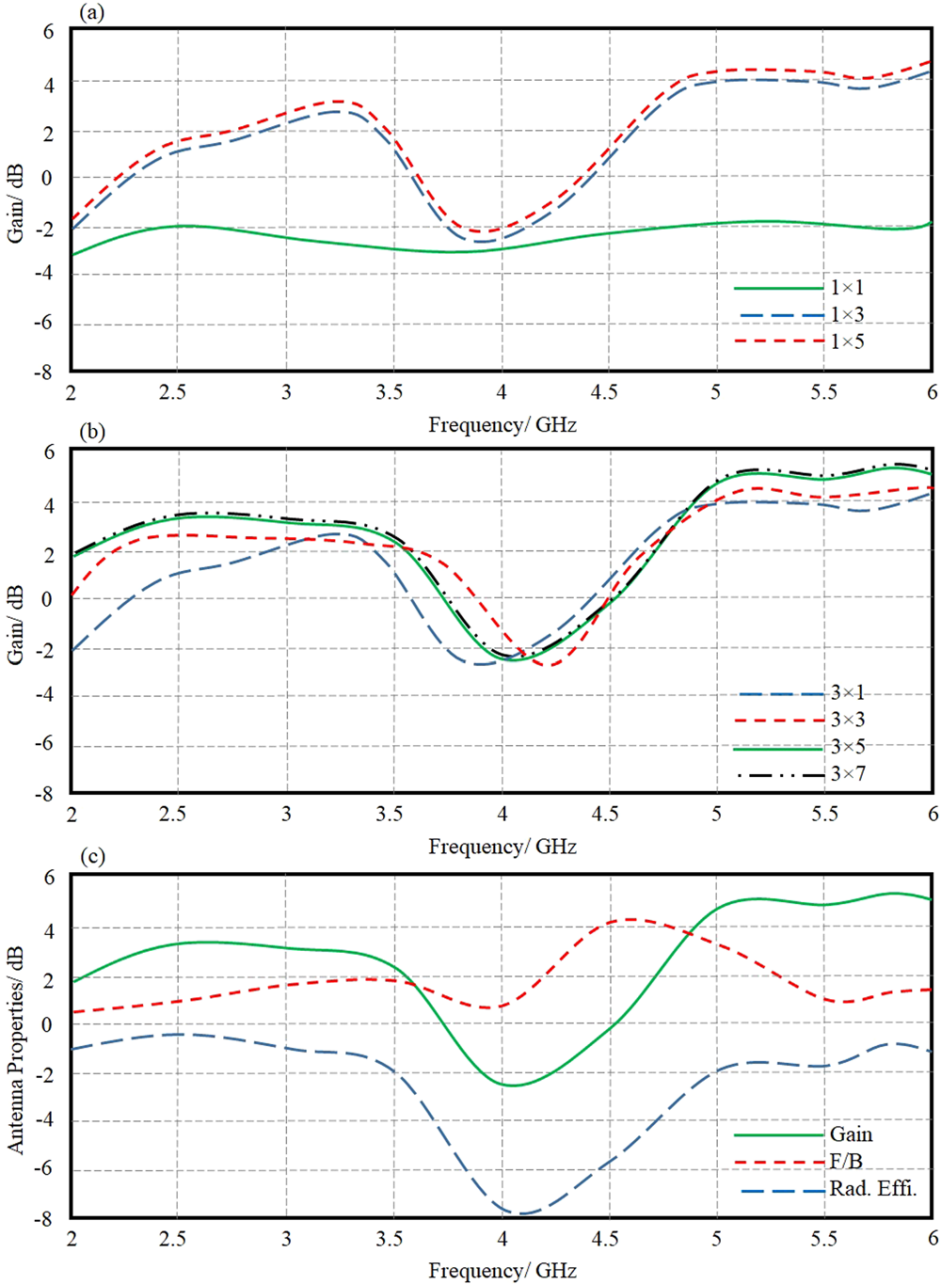
Antenna performance; (**a**) Gain spectra based columns parametric study, (**b**) Gain spectra based rows parametric study, and (**c**) the proposed antenna performance based 3 × 5 MTM array in terms of gain, F/B, Radiation Efficiency spectra.

Now, a parametric study using CST MWS is conducted to realize the best antenna location with respect to the proposed MTM array. In this study, the antenna location (S) is changed from 1 mm, 5 mm, to 9 mm. It is found that the antenna shows the maximum gain at S = 5 mm as shown in Fig. 13(a). This location agrees with the obtained results from the ray tracing analysis as presented in Fig. 13(b,c) at 2.45 GHz and 5.8 GHz, respectively. Nevertheless, the effect of the proposed MTM unit cell array periodicity on the proposed antenna gain is involved in this section. Odd array indexes only are considered in the proposed parametric study to ensure the centralized MTM unit cell location in front of the antenna center to achieve Gaussian beam distribution^23^. For this, the periodicity along the array columns is conducted as 1 × 1, 1 × 3, and 1 × 5 as seen in the gain spectra in Fig. 14(a). It is found that the proposed MTM unit cell provides a significant change after introducing three columns; however, there is no significant change after increasing the columns number to five. Therefore, the next parametric study is conducted on the rows with fixing the number of columns at three. In such case, the effects of rows increase is realized through changing the array from 3 × 1, 3 × 3, 3 × 5, and 3 × 7 as presented in Fig. 14(b). It is observed that increasing the rows number more than five has no significant effect on the antenna gain; that encouraged the author to consider the optimal number of rows is five. Finally, the optimal arrived antenna design with 3 × 5 MTM array performance in terms of gain, front to back ratio (F/B), and radiation efficiency spectra are evaluated and presented in Fig. 14(c) at the best antenna location from the MTM array. The gain enhancement is achieved significantly due to the ability of the proposed MTM to match between the electromagnetic aperture impedance of the antenna and the free space impedance.

## Measurement Validation and Discussion

**Figure 15:**
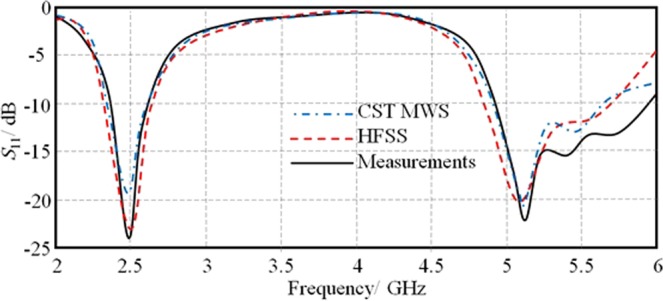
Comparison between measured and simulated *S*_11_ results.

**Figure 16:**
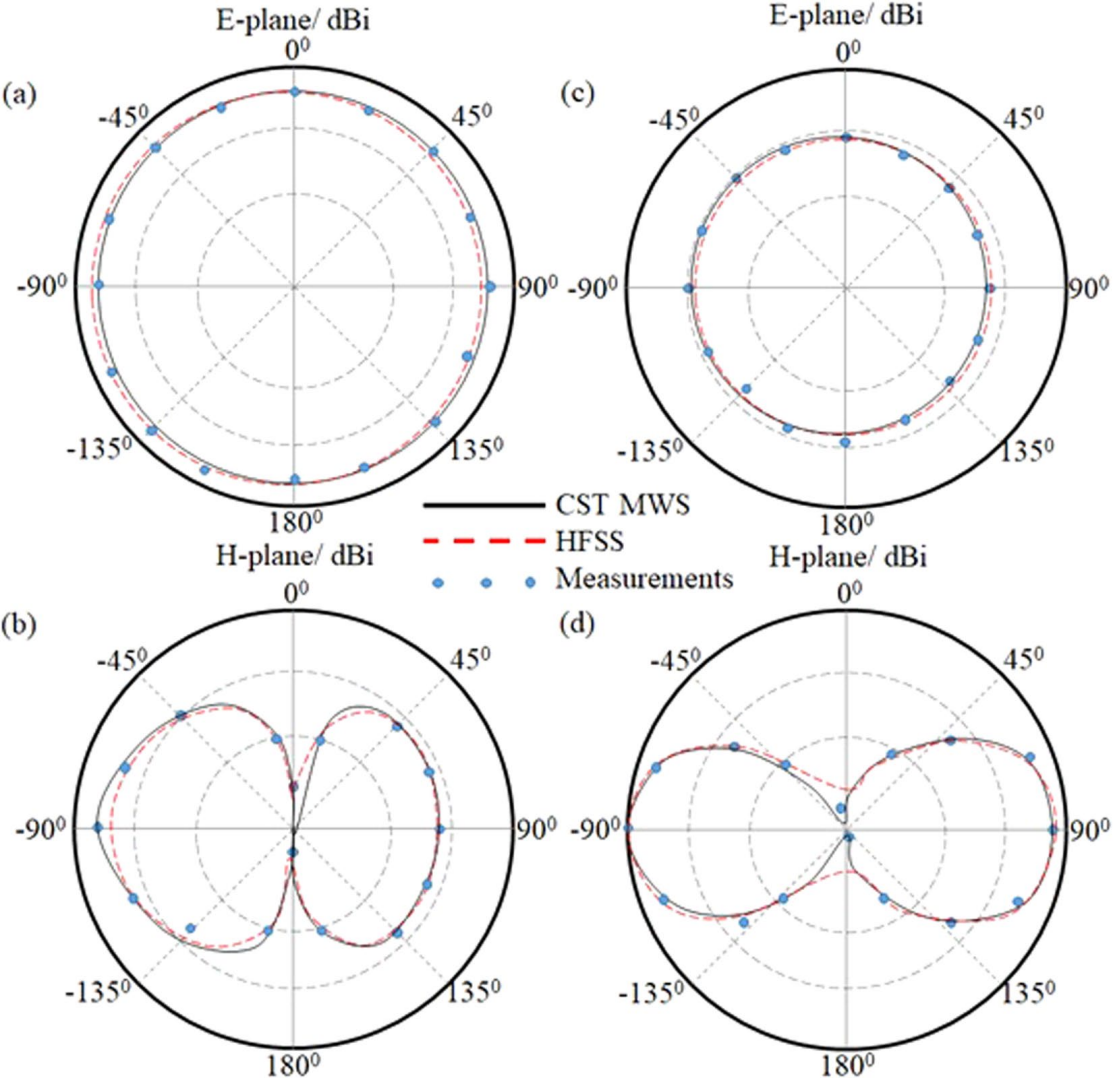
Comparison between measured and simulated far-field radiation patterns; (**a**,**b**) *E*-plane and *H*-plane, respectively at 2.45 GHz, while, (**c**,**d**) *E*-plane and *H*-plane, respectively at 5.8 GHz.

After evaluating the antenna performance using numerical analysis of CST MWS simulations, HFSS software package is invoked for validation before applying the measurements in terms of *S*_11_ and radiation patterns. The *S*_11_ measurement is conducted with HP 8720A VNA and the radiation patterns are measured inside a microwave anechoic chamber. It is found that the proposed antenna shows an excellent matching around 2.45 GHz and 5.8 GHz that suits the Wi-Fi applications as seen in Fig. 15. The comparison between the simulated and measured results reviles a less than 0.2% difference. The measured radiation patterns are performed at 2.45 GHz and 5.8 GHz then compared against the simulated results; it is found an obvious matching between the obtained results. Figure 16 shows the measured radiation patterns at both bands of interest in the *E*-plane and *H*-plane. From the measured radiation patterns, it is found that the realized gains of 2.6 dBi and 4.8 dBi at 2.45 GHz and 5.8 GHz, respectively. It is demonstrated an excellent agreement in terms of the gain pattern with less than 0.1% difference between simulated results and measurements. Furthermore, it is found that the angle of refraction of the emerged beam is diffracted to 18.6° that almost agrees with the obtained results from the ray tracing assumptions.

## Conclusion

The feasibility of using the palm fibers in the microwave industry is investigated in this paper for a novel application relative to the printed circuit antennas. A printed dipole antenna-based MTM structure for Wi-Fi band applications are exemplified for this study due to their potential importance. The substrate is prepared from mixing IPTR with NONP in PE hostel based on 3.4:0.1:6.5 mixture ratio. The electromagnetic constitutive parameters are computed based on experimental measurements from a T-stub resonator at the frequency band of interest to be used in the numerical design and analysis. The obtained *ε*_r_ and *μ*_r_ are found to be around 3.106-j0.0314 and 1.548-j0.0907, respectively. The MTM structure is printed on the INP substrate based on SNPCI using IJPT, while, the dipoles are fabricated from mounting a copper tape layer on each side. The antenna performance is analyzed numerically using CST MWS and HFSS simulations in terms of *S*_11_ and radiation patterns. After manufacturing the antenna prototype, the antenna is tested experimentally and compared to the obtained numerical results. It is found that the proposed antenna shows two frequency bands around 2.45 GHz and 5.8 GHz with gains of 2.6 dBi and 4.8 dBi, respectively, to show excellent agreements with the simulated results.

## References

[1] Caloz, C. & Itoh, T. Electromagnetic metamaterials: transmission line theory and microwave applications. (1^st^ ed.) *John Wiley & Sons*, 176–187 (2005).

[2] Bo L, Y. *et al*. Transmission-type 2-bit programmable metasurface for single-sensor and single-frequency microwave imaging. *Sci. Rep*., **6** (2016). 10.1038/srep23731 PMC4812328 27025907

[3] Dong Y, Itoh T (2012). Metamaterial-Based Antennas. Proc. IEEE.

[4] Chen, K. *et al*. Geometric phase coded metasurface: from polarization dependent directive electromagnetic wave scattering to diffusion-like scattering. *Sci. Rep*., **6** (2016). 10.1038/srep35968 PMC5075887 27775064

[5] Wan, X., Qing Qi, M., Yi Chen, T. & Jun Cui, T. Field-programmable beam reconfiguring based on digitallycontrolled coding metasurface. *Sci. Rep*., **6** (2016). 10.1038/srep20663 PMC4748258 26861110

[6] Zhu, B. O. *et al*. Dynamic control of electromagnetic wave propagation with the equivalent principle inspired tunable metasurface. *Sci. Rep*., **4** (2014).

[7] Zhang, Y., Wang, H., Liao, D. & Fu, W. Phase-tuning Metasurface for Circularly Polarized Broadside Radiation in Broadband. *Sci. Rep*. **8** (2018). 10.1038/s41598-018-21393-y PMC5813007 29445198

[8] Yuan, Y. *et al*. Complementary transmissive ultra-thin meta-deflectors for broadband polarization-independent refractions in the microwave region. *Phot. Rese*. **7** (2019).

[9] Pereda, A. T. *et al*. Experimental Validation of a Ku-Band Dual-Circularly Polarized Metasurface Antenna. *IEEE Tran. on Ant. and Prop*., **66** (2018).

[10] Jiang, Z. H., Brocker, D. E., Sieber, P. E., Werner, D. H. A Compact, Low-Profile Metasurface-Enabled Antenna for Wearable Medical Body-Area Network Devices. *IEEE Tran. on Ant. and Prop*, **62** (2014).

[11] Elwi, T. A. Novel UWB Printed Metamaterial Microstrip Antenna based Organic Substrates for RF-Energy Harvesting Applications. *AEU-Int J Electron C* **101**, 44–53 (2019).

[12] Elwi, T. A. *et al*. Multi-walled carbon nanotube-based RF antennas. *Nanotechnology*, **21** (2009). 10.1088/0957-4484/21/4/045301 20009173

[13] Elwi AT, Khudhayer WJ (2013). A passive wireless gas sensor based on microstrip antenna with copper nanorods. Prog. Electromagn. Res. B.

[14] Elwi, T. A. A miniaturized folded antenna array for MIMO applications. *Wirel. Pers. Commun*., 1–13 (2017).

[15] Elwi TA, Hamed MM, Abbas Z, Elwi MA (2014). On the performance of the 2D planar metamaterial structure. AEU-Int J Electron C.

[16] Shabdin, M. K., Mohamed-Shariff, A. R., Azlan-Johari, M. N., Saat, N. K. & Abbas, Z. A. Study on the oil palm fresh fruit bunch (FFB) ripeness detection by using Hue, Saturation and Intensity (HSI) approach. *IOP Conf. Ser.: Earth Environ. Sci*., **37** (2016).

[17] Mohassieb SA, Kirah K, Dörsam E, Khalil ASG, El-Hennawy HM (2017). Effect of silver nanoparticle ink drop spacing on the characteristics of coplanar waveguide monopole antennas printed on flexible substrates. IET MAP.

[18] Balanis, C. A. Antenna theory: Analysis and design. *John Wiley & Sons*, (3^rd^ ed.), 1036–1043 (2012).

[19] CST MWS, https://www.cst.com (2016).

[20] Wadell, B. C. Transmission line design handbook. *Artech House Antenna and Propagation Library*, 30–45 (1991).

[21] Shabdin MK (2016). A study on the oil palm fresh fruit bunch (FFB) ripeness detection by using Hue, Saturation and Intensity (HSI) approach. IOP Conf. Ser.: Earth Environ. Sci..

[22] Bahl, I. Lumped elements for RF and microwave circuits. *Artech House microwave library*, *Boston London*, 23–33 (2003).

[23] Elwi TA, Al-Rizzo HM, Rucker DG, Song F (2009). Numerical simulation of a UC-PBG lens for gain enhancement of microstrip antennas. Inter. Jour. of RF and Micro. Comp. Aid. Eng..

[24] Al-Naiemy Y, Elwi TA, Khaleel HR, Al-Rizzo HM (2012). A systematic approach for the design, fabrication and testing of microstrip antennas using ink-jet printing technology. ISRN Comm. and Net..

[25] HFSS, http://www.ansoft.com (2016).

[26] Albrecht A, Retzker A, Plenio MB (2014). Testing quantum gravity by nanodiamond interferometry with nitrogen-vacancy centers. Phys. Rev. A.

